# Dataset of NIR, MIR and FIR spectroscopy of fuels in maritime cases and biodiesel–diesel blends B7 and B10 from Malaysia

**DOI:** 10.1016/j.dib.2024.110615

**Published:** 2024-06-10

**Authors:** Mohd Rashidi Abdull Manap, Haziq Izwan Baharim, Nur Azalina Ajmahera Shamsudin, Ahmad Faridi Ferdaus

**Affiliations:** a Department of Chemistry, Faculty of Science, Universiti Putra Malaysia, 43400 Serdang, Selangor, Malaysia; b Ibu Pejabat Agensi Penguatkuasaan Maritim Malaysia, 4-11, One Square IOI, IOI Resort, 62502 Putrajaya, Malaysia

**Keywords:** ATR-FTIR, B7, B10, Biodiesel, Crime, Enforcement

## Abstract

The dataset contains Fourier-transform infrared (FTIR) spectroscopic analysis of fuels in maritime cases and biodiesel–diesel blends B7 and B10 from Malaysia. Fuels in maritime cases were donated by Agensi Penguatkuasaan Maritim Malaysia (APMM) in March 2023. The crime-related oil samples originated from maritime crime scenes located within Terengganu and Johor, Malaysia. Meanwhile, B7(DE5) and B10(D0) samples were obtained from pump stations in 2021. They are fuels used in Malaysian transportation system. The FTIR analysis was acquired in the full regions of FTIR (6000–80 cm^−1^) which are near-infrared (NIR), mid-infrared (MIR), and far-infrared (FIR). The IR spectra were recorded using Bruker Invenio-R (Universiti Putra Malaysia) spectrometer equipped with attenuated total reflection (ATR) (2 mm) diamond with an accumulation of 64 scans at a spectral resolution of 4 cm^−1^. Spectral analysis was carried out by OPUS 8.7.41. The data highlights the potential of NIR, MIR, and FIR spectroscopy as a powerful tool for forensic analysis in maritime crime investigations. This includes the potential of utilizing the Hierarchical Clustering Analysis (HCA) to discriminate between type of fuels in forensic cases.

Specifications Table

**Table:** 

Subject	Spectroscopy
Specific subject area	Fourier Transform Infrared (FT-IR)
Type of data	Figures with peak labeling. Raw data in 0.0 format Experimental file in .xpm format
Data collection	FT-IR spectra were acquired employing a Bruker Invenio-R (Universiti Putra Malaysia) spectrometer equipped with attenuated total reflection (ATR) (2 mm) diamond with an accumulation of 64 scans at a spectral resolution of 4 cm^−1^ and carried out by OPUS 8.7.41.
Data source location	Institution: Department of Chemistry, Faculty of Science, Universiti Putra Malaysia Country: Malaysia Latitude and longitude and GPS coordinates, for collected samples/data: 2°59′28.7″N 101°42′29.5″E
Data accessibility	Repository name: Mendeley data Direct URL to data: Manap, Mohd Rashidi Abdull; BAHARIM, HAZIQ IZWAN (2024), “Experimental data of crime-related fuel in Johor and Terengganu, Malaysia”, Mendeley Data, V1 Data identification number: 10.17632/kwm6bf95yt.1 Direct URL to data: https://data.mendeley.com/datasets/kwm6bf95yt/1 Instructions for accessing these data: The fuel spectra are provided in the Opus format. Click on the appropriate URL above to download the 0.0 file. Repository name: Manap, Mohd Rashidi Abdull; BAHARIM, HAZIQ IZWAN; SHAMSUDIN, NUR AZALINA AJMAHERA (2024), “Experimental raw data of B7 and B10”, Mendeley Data, V1 Data identification number: doi: 10.17632/d7sx7bf2hk.1 Direct URL to data: https://data.mendeley.com/datasets/d7sx7bf2hk/1 Instructions for accessing these data: The fuel spectra are provided in the Opus format. Click on the appropriate URL above to download the 0.0 file. Repository name: Manap, Mohd Rashidi Abdull (2024), “Experiment files and measurement parameters for Bruker Invenio-R”, Mendeley Data, V1, Data identification number: 10.17632/rp8nthpx4f.2 Direct URL to data: https://data.mendeley.com/datasets/rp8nthpx4f/1 Instructions for accessing this data: The experimental file is provided in the Opus format. Click on the appropriate URL above to download the .xpm file.

## 1 Value of the Data

• These analyses offered deep insights into the characteristics of the recent crime-related fuels in 2023.
• These analyses provide deep insights into the characterization of the 30 samples of B7 and B10 fuel.
• The data may be used for the characterization of the 15 crime-related samples in Johor and Terengganu, Malaysia.
• The data may be used for the characterization of B7 and B10 fuel samples.
• Chemists in the forensic industry and research areas have the advantage of the easily accessing information.
• The data may be used to identify either the fuel samples are originated from Malaysia or foreign country.
• The data may be used to identify the type of crime-related fuel samples after clustering method of HCA.
• Experimental data are useful for the validation of spectra with quantum and mechanical calculations.

## 2 Background

These datasets (15 samples) [1] originated from the crime-related smuggling of oil in Terengganu and Johor, Malaysia, as obtained from Agensi Penguatkuasaan Maritim Malaysia (APMM). Meanwhile, the datasets [2] cover 30 fuel samples of B7 and B10 obtained from two major cities in Malaysia and the sample collection was performed directly from the fuel pump nozzle. Authors will utilize these data to for multivariate analysis in different FTIR spectral region which are near-infrared, mid-infrared, and far-infrared spectroscopy. Additionally, the spectroscopic dataset contributes valuable information to the characterization of spectra within the near-infrared (NIR), mid-infrared (MIR), and far-infrared (FIR) spectroscopy regions. In future data analysis, chemometric techniques such as Hierarchical Clustering Analysis (HCA) with the utilization of effect of spectral range will be employed to differentiate between various fuel blends, specifically B7 and B10. Additionally, these techniques will be used to distinguish between fuel samples collected in Malaysia and forensic samples. Also, for the spectral library of fuels in Malaysia.

## 3 Data Description

Table 1 shows 15 samples of crime-related fuel donated by the Agensi Penguatkuasaan Maritim Malaysia (APMM). These samples were seized from criminal activities in Johor and Terengganu. In February 2023, APMM successfully retrieved oil from the several boats and lorry trucks in Johor and Terengganu. The lot numbers assigned by APMM have been modified to facilitate the researchers understanding and project requirements.

**Table 1:** List of 15 crime-related fuel samples donated by Agensi Penguatkuasaan Maritim Malaysia (APMM) in Johor and Terengganu, Malaysia that acquired from tank of boats and trucks.

Sample	Location	UPM Lot number	APMM Lot number	Type of vehicle	Sampling Date (D/M/Y)	Time	Remarks
1	Desaru	OIL_SLDJ_23_819_01	NCY 9922	Truck	7/2/2023	1230AM	Under investigation
2	Desaru	OIL_SLDJ_23_819_02	MT IBU	Boat	6/2/2023	1145AM	Under investigation
3	Desaru	OIL_SLDJ_23_819_03	BER 4521	Truck	7/2/2023	1240AM	Under investigation
4	Desaru	OIL_SLDJ_23_819_04	NCY 9922	Truck	7/2/2023	1230AM	Under investigation
5	Desaru	OIL_SLDJ_23_819_05	BER 4251	Truck	7/2/2023	1250AM	Under investigation
6	Pantai Timur	OIL_PTT_23_203_01	PAF 4903	Truck	3/2/2023	1030AM	Disposed
7	Pantai Timur	OIL_PTT_23_203_02	KNF 7909	Truck	3/2/2023	1040AM	In disposal process
8	Pantai Timur	OIL_PTT_23_203_03	KG 2025 TS	Boat	3/2/2023	1050AM	In disposal process
9	Pantai Timur	OIL_PTT_23_203_04	KG 2026 TS	Boat	3/2/2023	1100AM	In disposal process
10	Pantai Timur	OIL_PTT_23_203_05	KG 94,785 TS	Boat	3/2/2023	1110AM	Disposed
11	Pantai Timur	OIL_PTT_23_203_06	KG 90,006 TS	Boat	3/2/2023	1120AM	In disposal process
12	Pantai Timur	OIL_PTT_23_203_07	BD 31,223 TS	Boat	3/2/2023	1130AM	In disposal process
13	Pantai Timur	OIL_PTT_23_203_08	BD 30,865 TS	Boat	3/2/2023	1140AM	In disposal process
14	Pantai Timur	OIL_PTT_23_203_09	BD 93,430 TS	Boat	3/2/2023	1150AM	Disposed
15	Pantai Timur	OIL_PTT_23_203_10	BD 30,129 TS	Boat	3/2/2023	1200AM	Disposed

As shown in Table 2, 10 samples of B7 (DE5) and 20 samples of B10 (D0) samples obtained from Mersing and Seri Kembangan, Malaysia. For each sample, the shipping point is included based on the last delivery order and they were supplied on the same month. The raw data files can be accessed at https://data.mendeley.com/datasets/kwm6bf95yt/1.

**Table 2:** List of B7(DE5) and B10(D0) samples obtained from Mersing and Seri Kembangan, Malaysia.

No	Company name	Product	Brand	Shipping point	Delivery date (D/M/Y)	Lot number	Sampling date (M/D/Y) and time (pm)
1	NURRAZZAK ENTERPRISE	INFINITI DIESEL	BHPetrol	NORTH PORT INSTALLATION TERMINAL	8/10/2021	DE5_BP1_433	10/6/2021 11:10
2	NURRAZZAK ENTERPRISE	BIO EURO 5 BIO DIESEL	BHPetrol	NORTH PORT INSTALLATION TERMINAL	8/10/2021	D0_BP1_433	10/6/2021 11:12
3	AZAMEGA ENTERPRISE	BIODIESEL B10 EURO 5	Petronas	PDB KVDT Fuel	4/10/2021	D0_PS1_433	10/5/2021 11:10
4	PADI EMAS PETROLEUM SDN. BHD.	EURO5 B7 POWERD W TECHROND/BULK	Caltex	PULAU INDAH TRM CML	1/10/2021	DE5_CX1_433	10/5/2021 12:02
5	PADI EMAS PETROLEUM SDN. BHD.	EURO5 B10 DIESEL W TECHROND/BULK	Caltex	PULAU INDAH TRM CML	3/10/2021	D0_CX1_433	10/5/2021 12:08
6	SRI KEMBANGAN SERVICE STATION	TURBO DIESEL E5	Petron (PMRMB)	KLANG VALLEY DIST	4/10/2021	DE5_PN1_433	10/5/2021 10:35
7	SRI KEMBANGAN SERVICE STATION	DIESEL MAX B10	Petron (PMRMB)	KLANG VALLEY DIST	4/10/2022	D0_PN1_433	10/5/2021 10:38
8	K.C.LIU AUTO CENTRE	DIESEL MAX B10	Petron (PFISB)	KLANG VALLEY DIST	4/10/2021	D0_PN2_433	10/6/2021 9:32
9	SERDANG RAYA ENTERPRISE	SHELL FUELSAVE DIESEL EURO 5 B10 MAINGRADE BULK	Shell	KLANG VALLEY PSP DEPOT	3/10/2021	D0_SL1_433	10/6/2021 10:10
10	BAKHTIAR ENTERPRISE	SHELL FUELSAVE DIESEL EURO 5 B10 MAINGRADE BULK	Shell	KLANG VALLEY PSP DEPOT	27/9/2021	D0_SL2_433	10/5/2021 9:59
11	ECAH ENTERPRISE	TURBO DIESEL E5	Petron (PMRMB)	KLANG VALLEY DIST	4/10/2021	DE5_PN3_433	10/7/2021 10:05
12	ECAH ENTERPRISE	DIESEL MAX B10	Petron (PMRMB)	KLANG VALLEY DIST	4/10/2021	D0_PN3_433	10/7/2021 10:07
13	MAZA INDAH ENTERPRISE	BIODIESEL B10 EURO 5	Petronas	PDB KVDT Fuel	7/10/2021	D0_PS2_433	10/7/2021 10:41
14	BE STATION	BIODIESEL B10 EURO 5	Petronas	PDB KVDT Fuel	4/10/2021	D0_PS3_433	10/5/2021 9:13
15	BE STATION	BIODIESEL B7 EURO5 PREMIUM	Petronas	PDB KVDT Fuel	28/9/2021	DE5_PS3_433	10/5/2021 9:14
16	PERNIAGAAN PROJEK CERGAS	SHELL FUELSAVE DIESEL EURO 5 B10 BULK	Shell	KLANG VALLEY PSP DEPOT	3/10/2021	D0_SL3_433	10/7/2021 11:18
17	PERNIAGAAN PROJEK CERGAS	SHELL FUELSAVE DIESEL EURO 5 B7 BULK	Shell	KLANG VALLEY PSP DEPOT	3/10/2021	DE5_SL3_433	10/7/2021 11:19
18	DEFT ONES RESOURCES	DIESEL MAX B10	Petron (PMRMB)	KLANG VALLEY DIST	7/10/2021	D0_PN4_433	10/7/2021 9:08
19	ZEENUN ENTERPRISE	BIODIESEL B7 EURO5 PREMIUM	Petronas	PDB KVDT Fuel	6/10/2021	DE5_PS4_433	10/6/2021 11:45
20	ZEENUN ENTERPRISE	BIODIESEL B10 EURO 5	Petronas	PDB KVDT Fuel	6/10/2021	D0_PS4_433	10/6/2021 11:47
21	SWEE HUP SDN. BHD.	EURO5 B10 DIESEL W TECHROND/BULK	Caltex	PULAU INDAH TRM CML	6/10/2021	D0_CX2_433	10/6/2021 10:32
22	TAHAMA SDN. BHD.	DIESEL MAX B10	Petron	PASIR GUDANG FUELS	11/9/2021	D0_PN5_868	9/11/2021 12:26
23	KPF NIAGA SDN. BHD.	B10 EURO 5 BIO DIESEL	BHPetrol	PUSAT PETROLEUM TANJUNG BIN	8/9/2021	D0_BP2_868	9/11/2021 14:04
24	CHI BROTHERS SERVICE STATION	TURBO DIESEL E5	Petron	PASIR GUDANG FUELS	10/9/2021	DE5_PN6_868	9/12/2021 9:51
25	CHI BROTHERS SERVICE STATION	DIESEL MAX B10	Petron	PASIR GUDANG FUELS	7/9/2021	D0_PN6_868	9/12/2021 10:11
26	KPF NIAGA SDN. BHD.	BIODIESEL B10 EURO 5	Petronas	PDB P GUDANG FUEL	10/9/2021	D0_PS5_868	9/11/2021 11:40
27	KPF NIAGA SDN. BHD.	BIODIESEL B7 EURO5 PREMIUM	Petronas	PDB P GUDANG FUEL	8/9/2021	DE5_PS5_868	9/11/2021 11:48
28	SEAH JIN KEE (MERSING) SDN. BHD	EURO5 B10 DIESEL WTECHROND/BULK	Caltex	PASIR GUDANG TRM CML	2/9/2021	D0_CX3_868	9/12/2021 11:07
29	MERSING PETROLEUM SDN. BHD.	B7 EURO 5 BIO DIESEL	BHPetrol	PUSAT PETROLEUM TANJUNG BIN	1/9/2021	DE5_BP3_868	9/12/2021 9:07
30	MERSING PETROLEUM SDN. BHD.	B10 EURO 5 BIO DIESEL	BHPetrol	PUSAT PETROLEUM TANJUNG BIN	1/9/2021	D0_BP3_868	9/12/2021 9:20

**Fig. 1:**
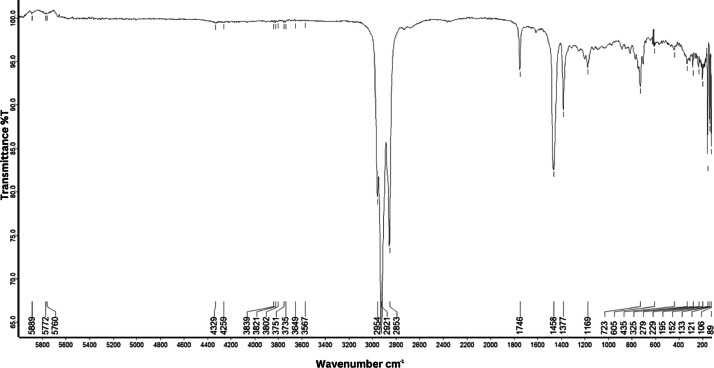
FT-IR for the OIL_SLDJ_819_01.

As shown in Fig. 1 until Fig. 45, the FT-IR spectra were acquired using a Bruker Invenio-R (Universiti Putra Malaysia) spectrometer equipped with an attenuated total reflection (ATR) (2 mm) diamond. The spectra acquisition involved 64 scans with a spectral resolution of 4 cm^−1^ and the spectra were processed using OPUS 8.7.41. The FT-IR spectra are also stored in the Mendeley depository. The transmittance spectra (in percentage transmittance,%T) were acquired for each sample between 80 and 6000 cm^−1^ covering near-infrared (NIR), mid-infrared (MIR), and far-infrared (FIR) spectra region. The crime-related fuel samples spectra from Johor are shown in Fig. 1 until Fig. 5. Meanwhile, crime-related sample spectra from Terengganu are shown in Fig. 6 until Fig. 15. The B7 sample spectra are shown in Fig. 16 until Fig. 25. Meanwhile, B10 sample spectra are shown in Fig. 26 until Fig. 45.

**Fig. 2:**
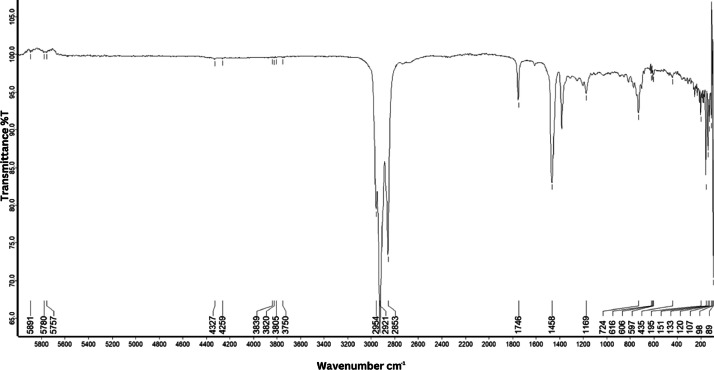
FT-IR for the OIL_SLDJ_23_819_02.

**Fig. 3:**
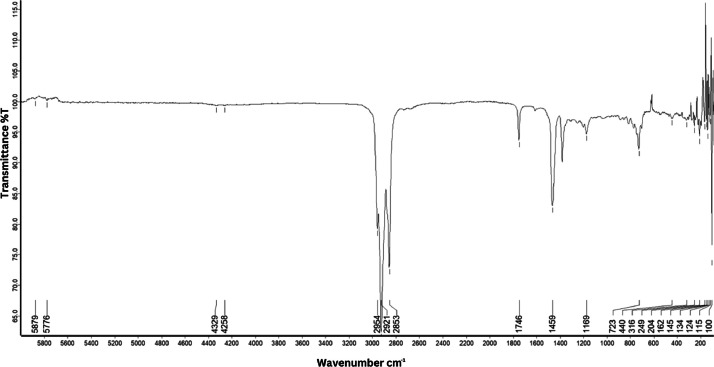
FT-IR for the OIL_SLDJ_23_819_03.

**Fig. 4:**
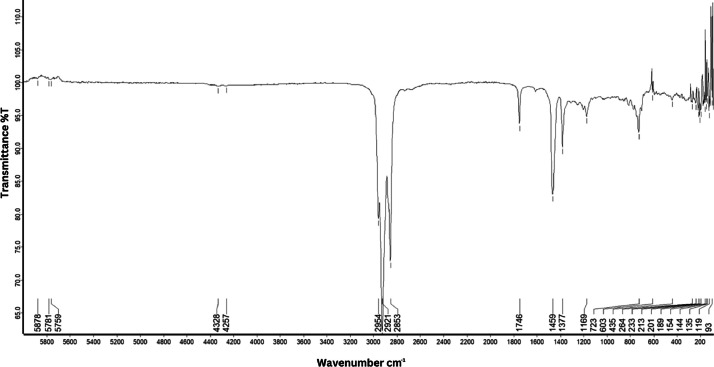
FT-IR for the OIL_SLDJ_23_819_04.

**Fig. 5:**
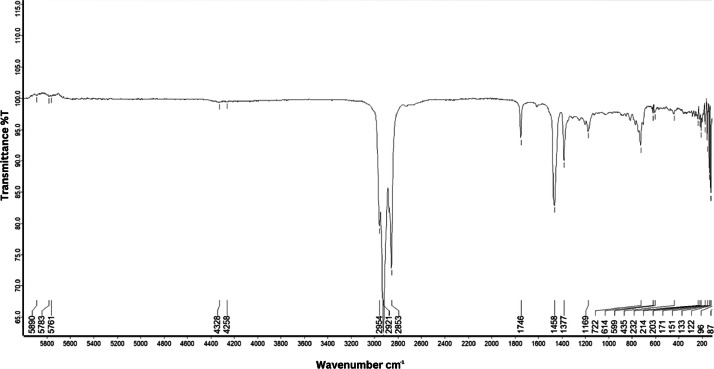
FT-IR for the OIL_SLDJ_23_819_05.

**Fig. 6:**
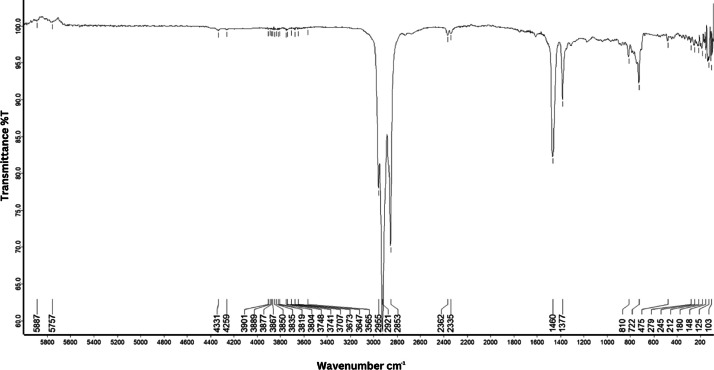
FT-IR for the OIL_PTT_23_203_01.

**Fig. 7:**
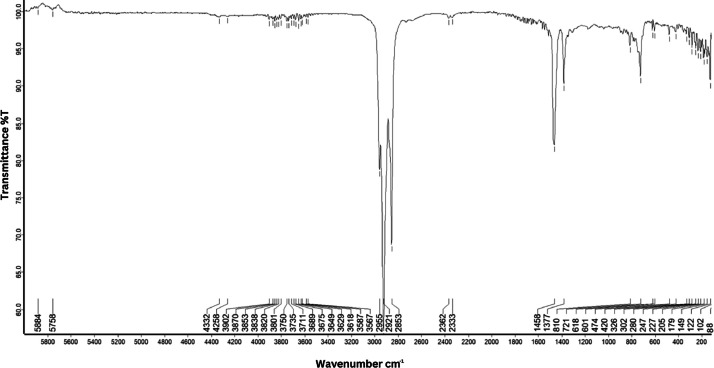
FT-IR for the OIL_PTT_23_203_02.

**Fig. 8:**
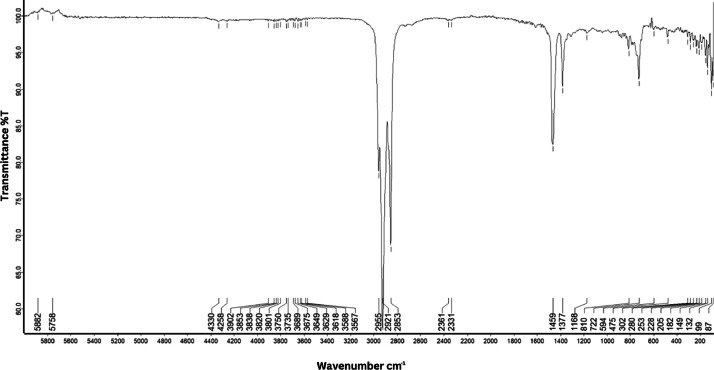
FT-IR for the OIL_PTT_23_203_03.

**Fig. 9:**
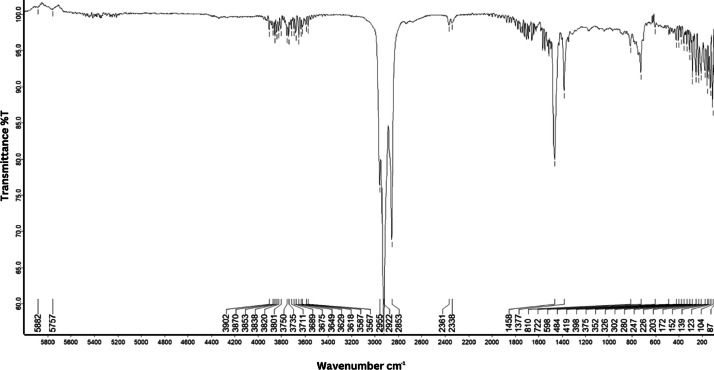
FT-IR for the OIL_PTT_23_203_04.

**Fig. 10:**
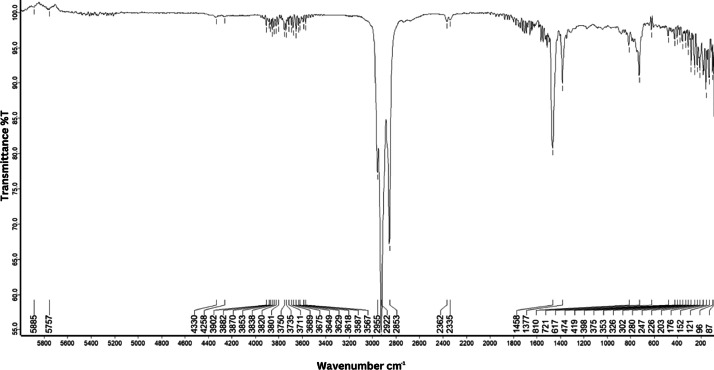
FT-IR for the OIL_PTT_23_203_05.

**Fig. 11:**
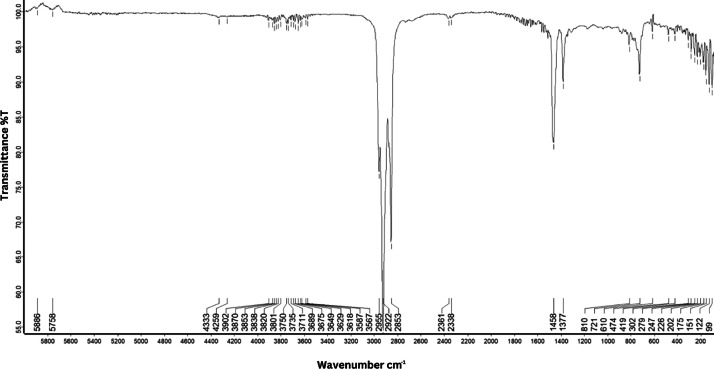
FT-IR for the OIL_PTT_23_203_06.

**Fig. 12:**
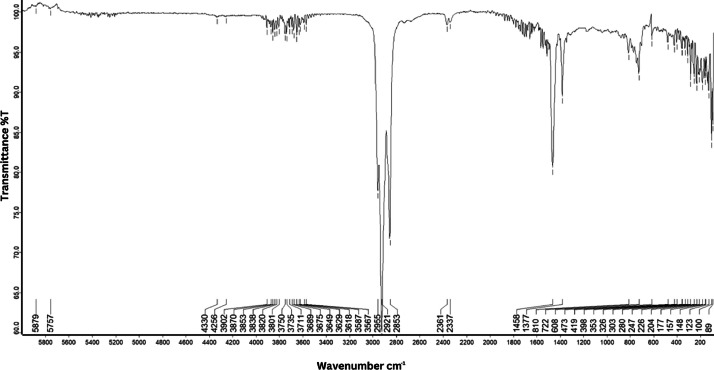
FT-IR for the OIL_PTT_23_203_07.

**Fig. 13:**
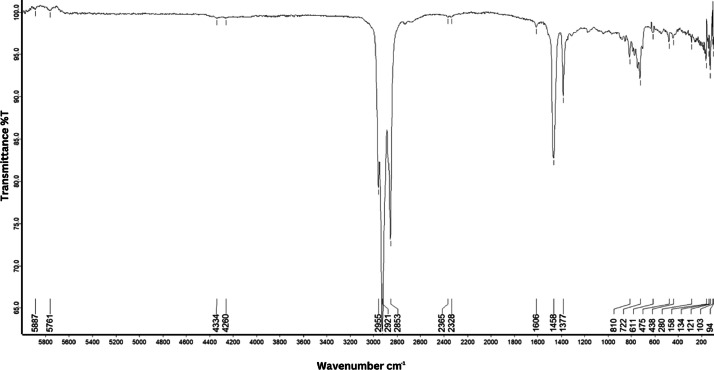
FT-IR for the OIL_PTT_23_203_08.

**Fig. 14:**
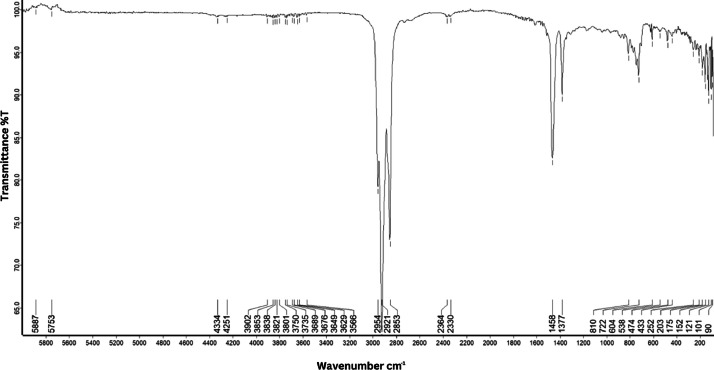
FT-IR for the OIL_PTT_23_203_09.

**Fig. 15:**
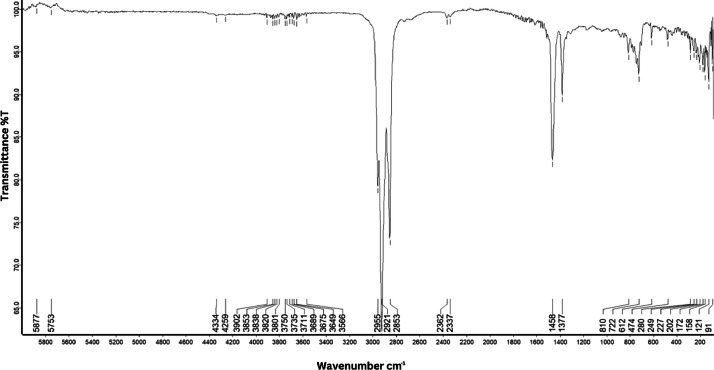
FT-IR for the OIL_PTT_23_203_10.

**Fig. 16:**
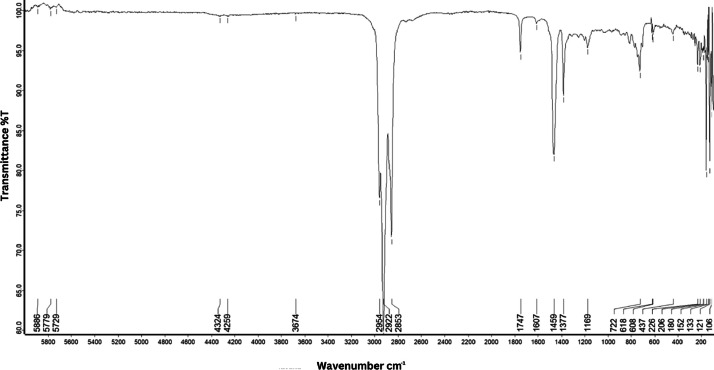
FT-IR spectra for the DE5_BP1_433.

**Fig. 17:**
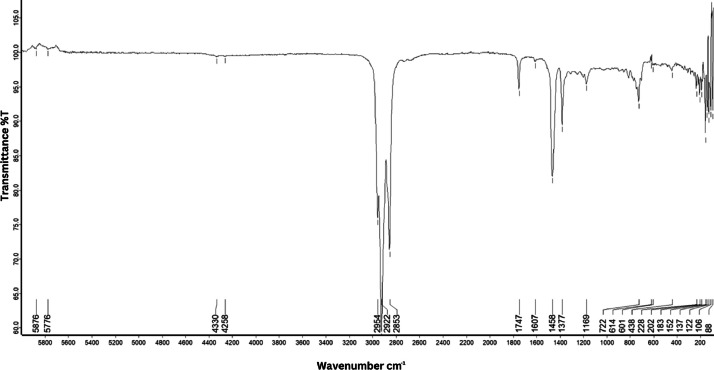
FT-IR spectra for the DE5_BP3_868.

**Fig. 18:**
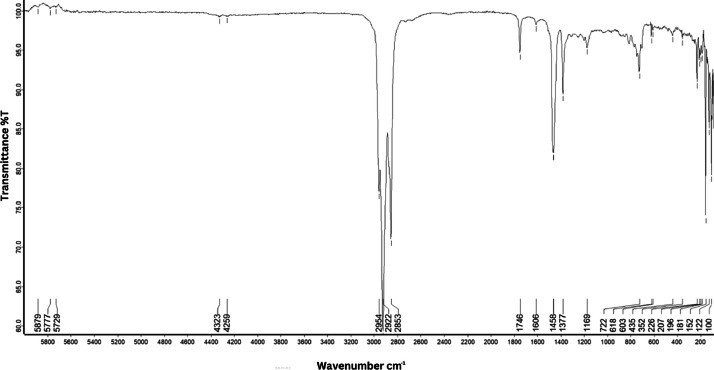
FT-IR spectra for the DE5_CX1_433.

**Fig. 19:**
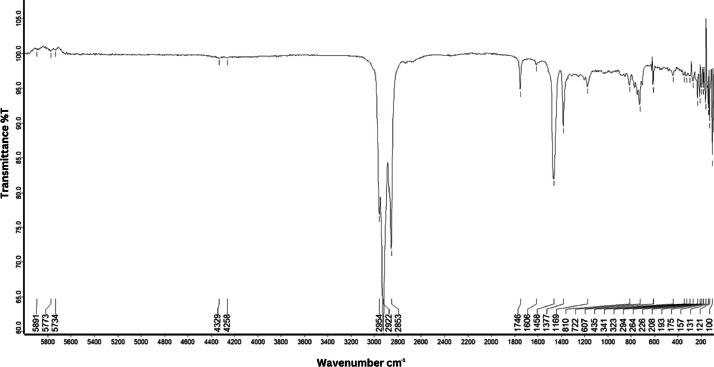
FT-IR spectra for the DE5_PN1_433.

**Fig. 20:**
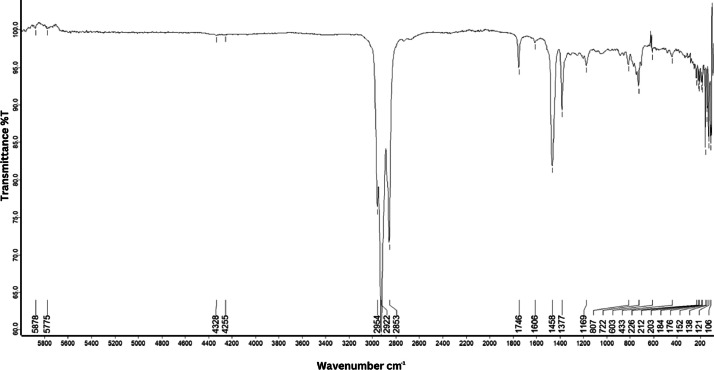
FT-IR spectra for the DE5_PN3_433.

**Fig. 21:**
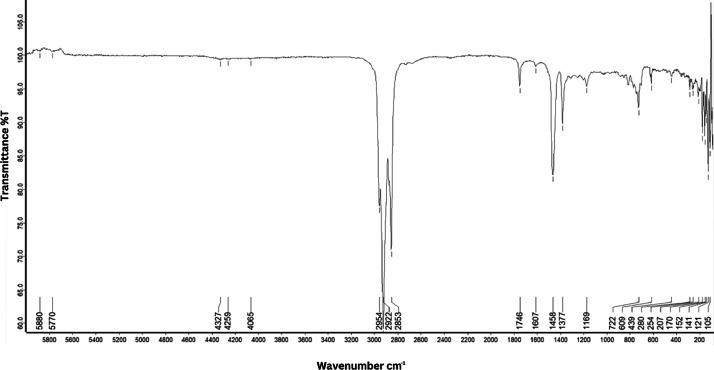
FT-IR spectra for the DE5_PN6_868.

**Fig. 22:**
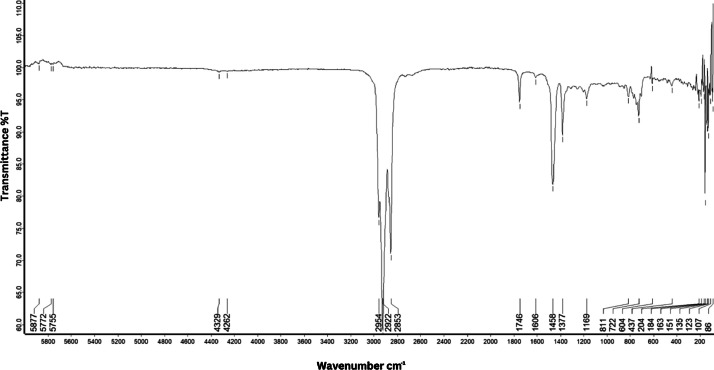
FT-IR spectra for the DE5_PS3_433.

**Fig. 23:**
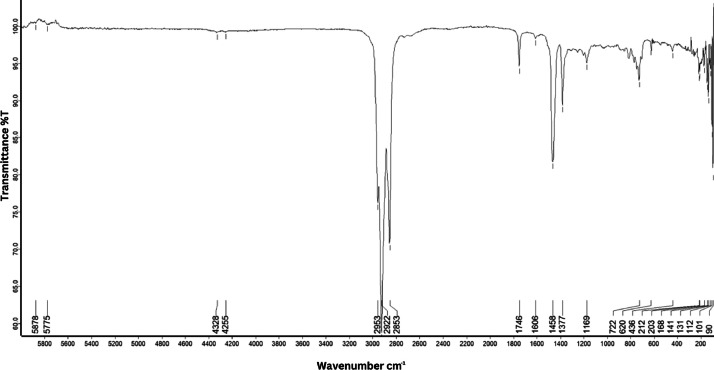
FT-IR spectra for the DE5_PS4_433.

**Fig. 24:**
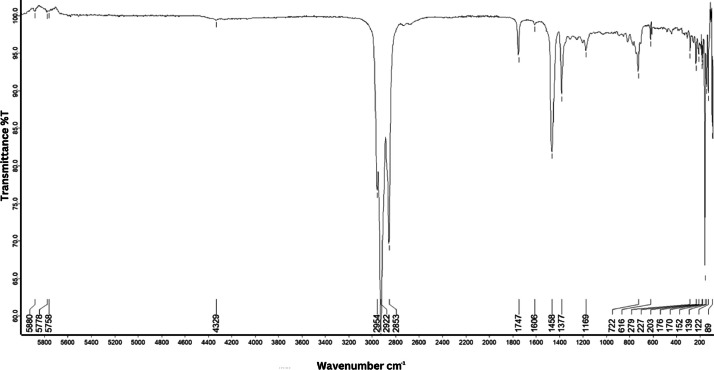
FT-IR spectra for the DE5_PS5_868.

**Fig. 25:**
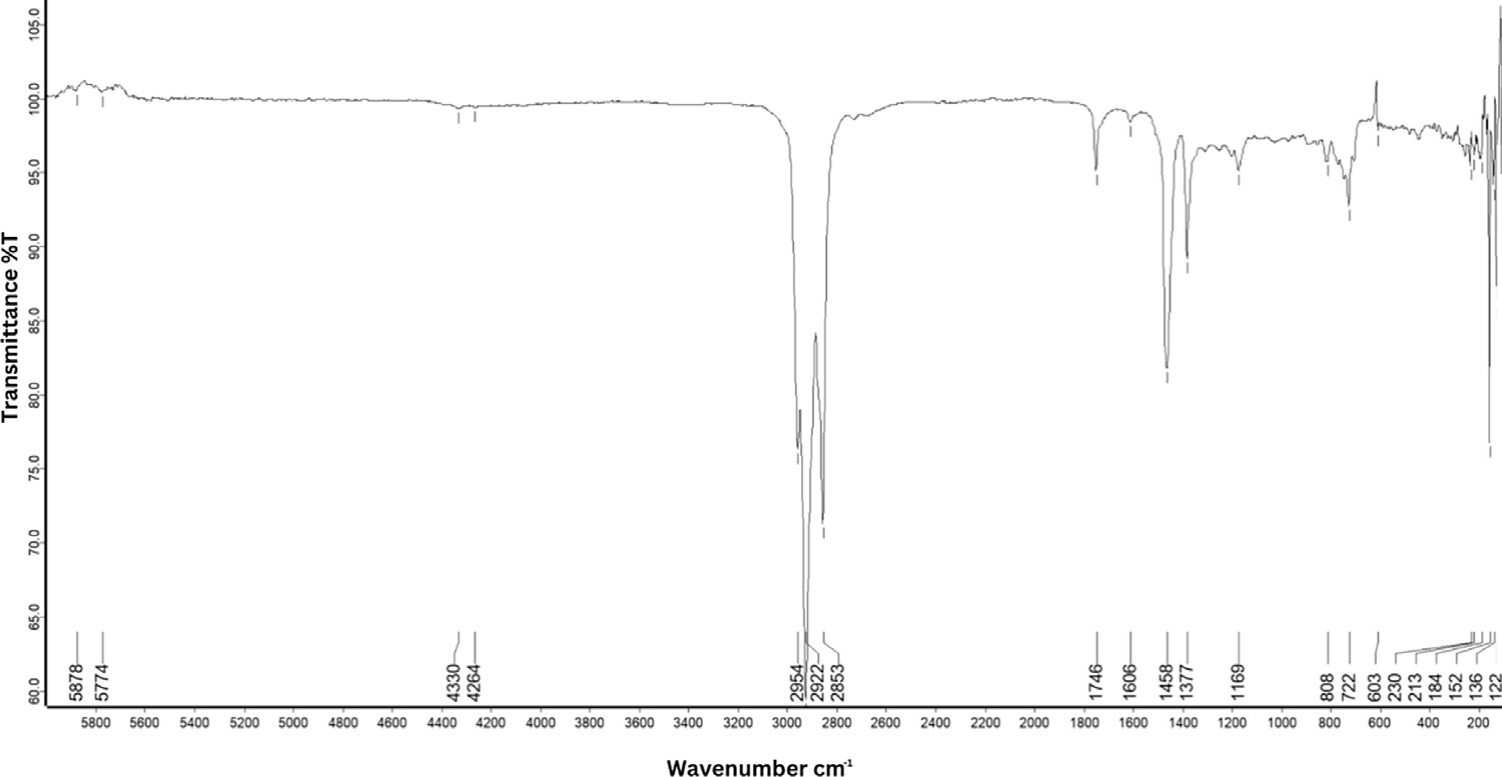
FT-IR spectra for the DE5_SL3_433.

**Fig. 26:**
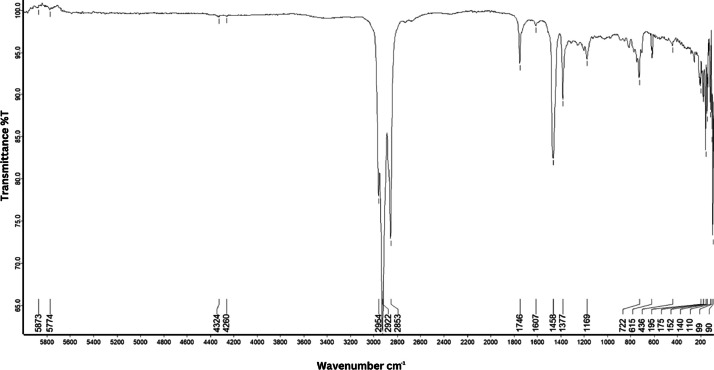
FT-IR spectra for the DO_BP1_433.

**Fig. 27:**
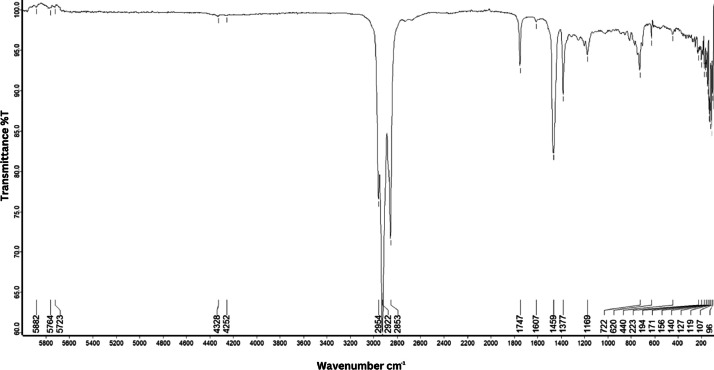
FT-IR spectra for the DO_BP2_868.

**Fig. 28:**
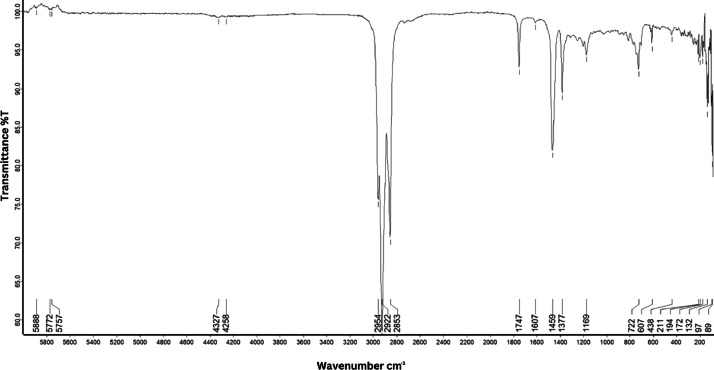
FT-IR spectra for the DO_BP3_868.

**Fig. 29:**
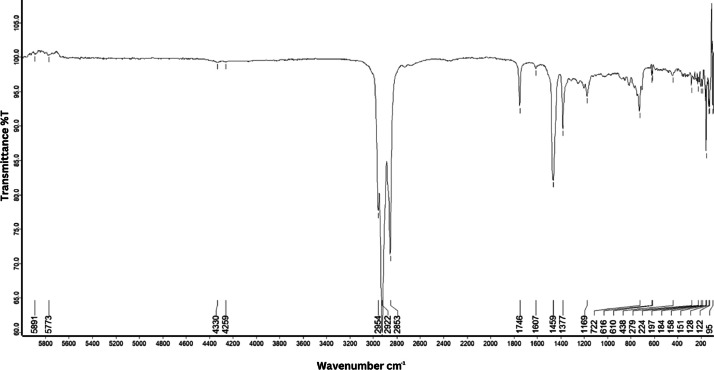
FT-IR spectra for the DO_CX1_433.

**Fig. 30:**
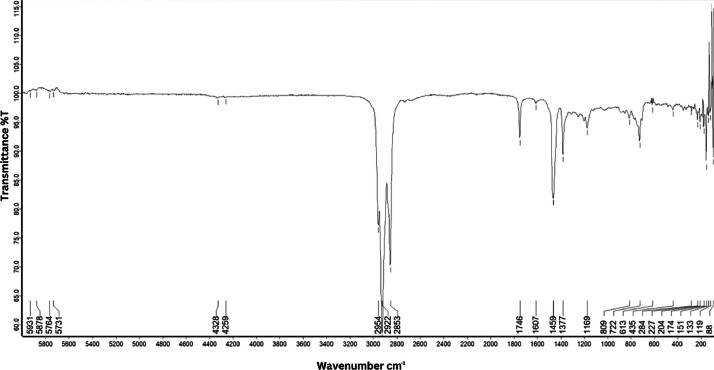
FT-IR spectra for the DO_CX2_433.

**Fig. 31:**
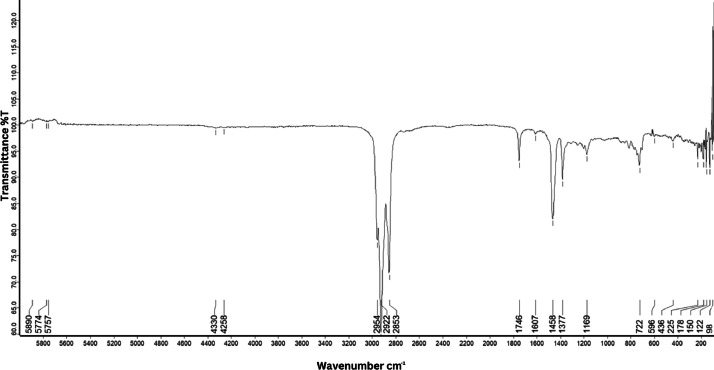
FT-IR spectra for the DO_CX3_868.

**Fig. 32:**
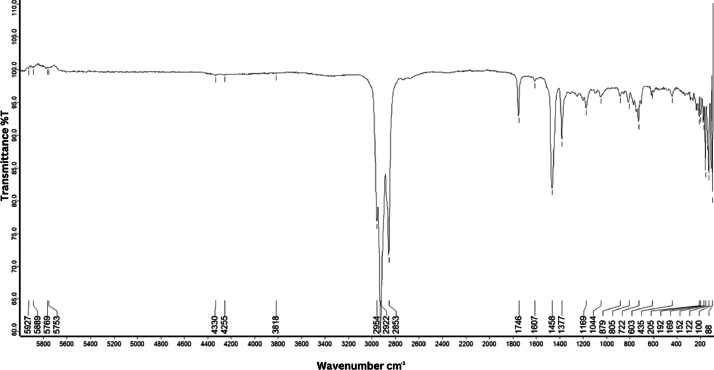
FT-IR spectra for the DO_PN1_433.

**Fig. 33:**
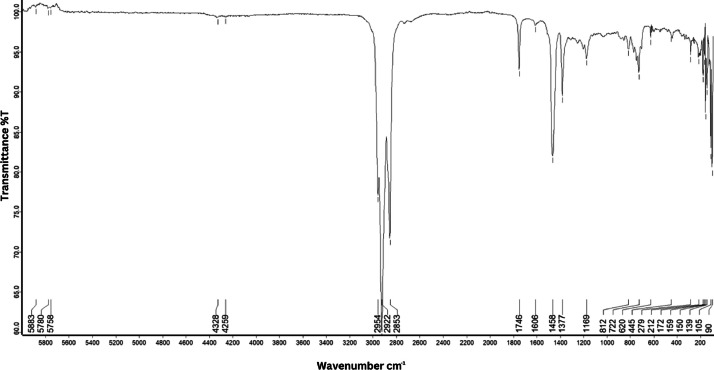
FT-IR spectra for the DO_PN2_433.

**Fig. 34:**
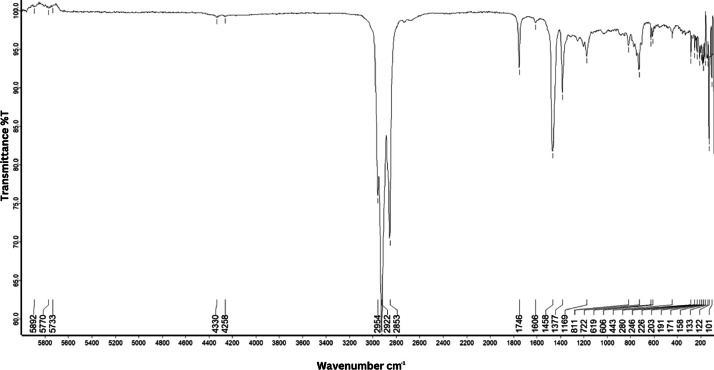
FT-IR spectra for the DO_PN3_433.

**Fig. 35:**
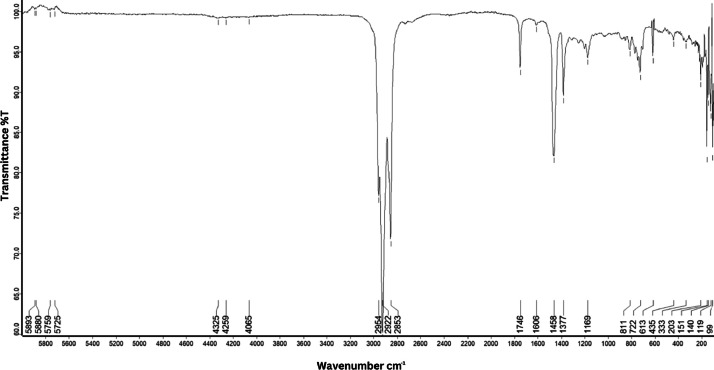
FT-IR spectra for the DO_PN4_433.

**Fig. 36:**
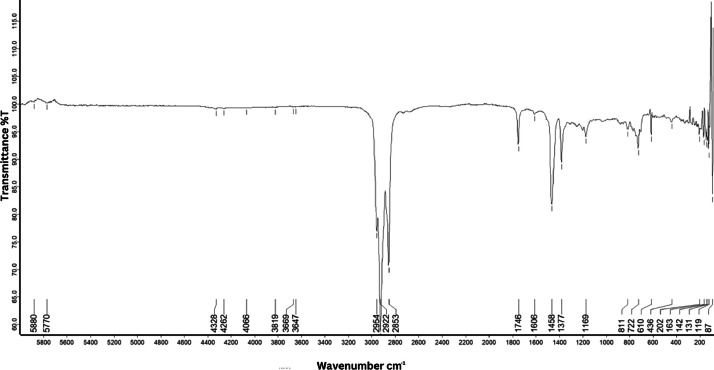
FT-IR spectra for the DO_PN5_868.

**Fig. 37:**
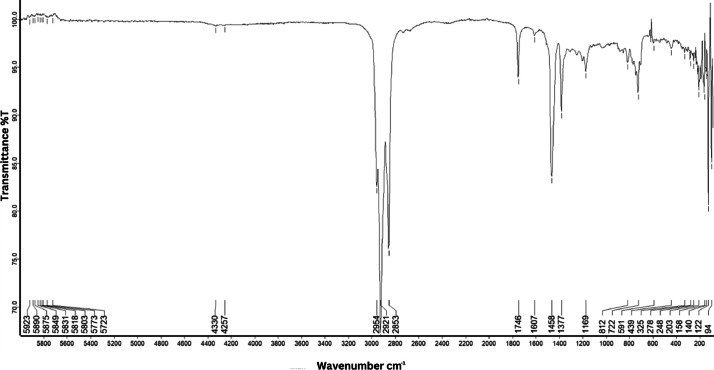
FT-IR spectra for the DO_PN6_868.

**Fig. 38:**
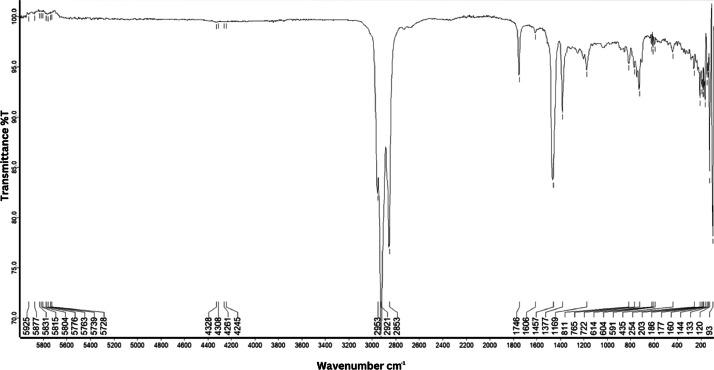
FT-IR spectra for the DO_PS1_433.

**Fig. 39:**
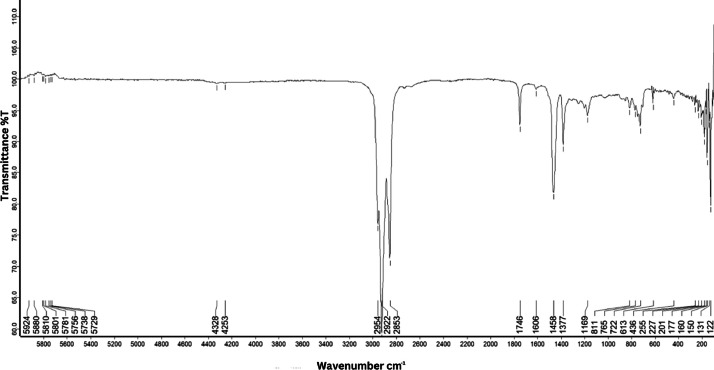
FT-IR spectra for the DO_PS2_433.

**Fig. 40:**
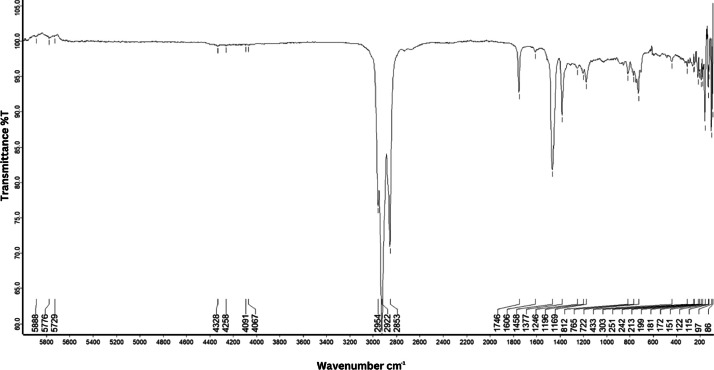
FT-IR spectra for the DO_PS3_433.

**Fig. 41:**
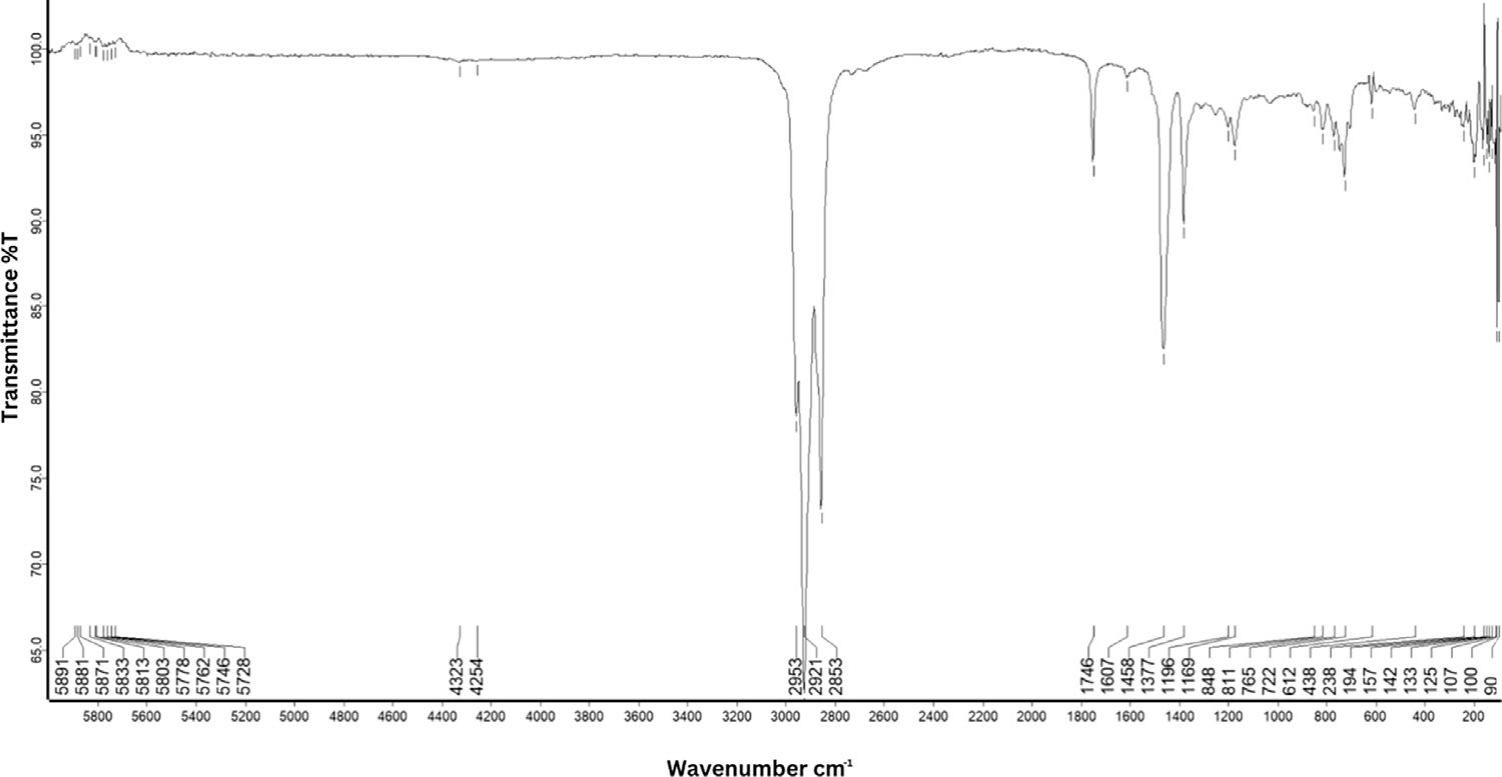
FT-IR spectra for the DO_PS4_433.

**Fig. 42:**
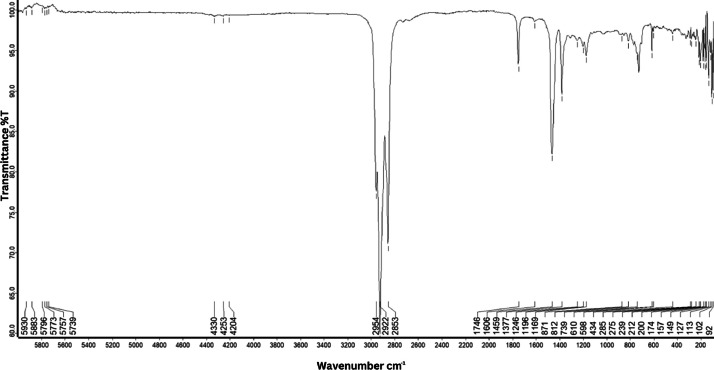
FT-IR spectra for the DO_PS5_868.

**Fig. 43:**
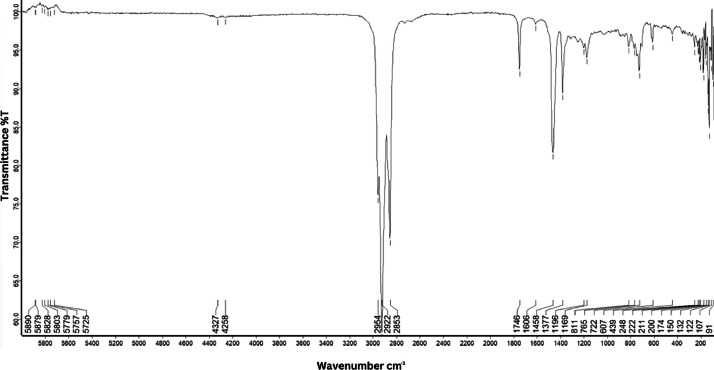
FT-IR spectra for the DO_SL1_433.

**Fig. 44:**
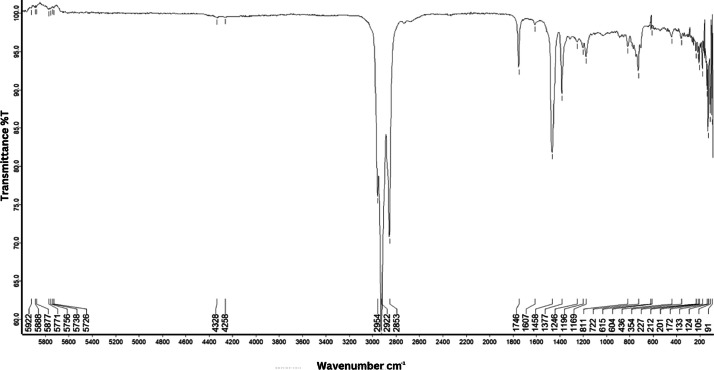
FT-IR spectra for the DO_SL2_433.

**Fig. 45:**
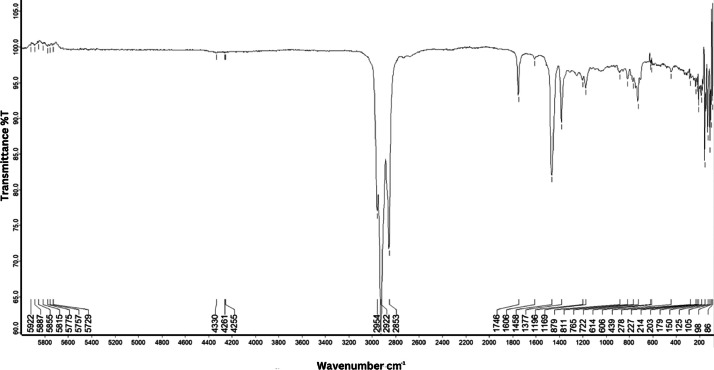
FT-IR spectra for the DO_SL3_433.

## 4 Experimental Design, Materials and Methods

At the beginning of the sampling process at the fuel pump station, cleaned and dried 1-l dark glass bottles were used, each labelled with a Lot Number for identification. To ensure the bottles were free of contaminants, the internal surfaces were rinsed with a small amount of the same fuel being sampled, and this initial wash was discarded. Subsequently, at least one liter of fuel was pumped directly from the fuel pump nozzle into the prepared bottles. To prevent sample adulteration from sunlight exposure, the storage bottles were wrapped in black plastic wrapping. The sealed samples were then transported to the UPM laboratory and stored at room temperature until they were ready for infrared (IR) analysis. The seals were only removed immediately prior to analysis to prevent any potential contamination. For this study, 30 biodiesel samples from Malaysia were analyzed. Upon receipt, the samples were assigned Lot Numbers by Agensi Penguatkuasaan Maritim Malaysia (APMM), which were subsequently modified to facilitate researcher understanding and to meet project requirements. Each sample was carefully documented and labelled according to the modified Lot Numbers. Proper precautions were maintained throughout the storage period to preserve the integrity of the samples. The dark glass bottles and black plastic wrapping provided protection from light, and the bottles remained sealed until the time of analysis (Figs. 2, 7 and 8).

For the forensic study, 15 samples of Illegal, Unreported, and Unregulated (IUU) fuel, seized by the (APMM) from criminal activities in Johor and Terengganu, Malaysia, were analyzed. These samples, obtained in March 2023, were retrieved by APMM in February 2023 from apprehended vessels. All collected samples were stored at room temperature in the UPM laboratory, with specific precautions taken to prevent contamination and degradation. The use of dark glass bottles and black plastic wrapping ensured minimal exposure to light, maintaining sample integrity. The sealed samples were only unsealed at the time of analysis. No sample pretreatment was applied to any samples before IR analysis.

**Fig. 46:**
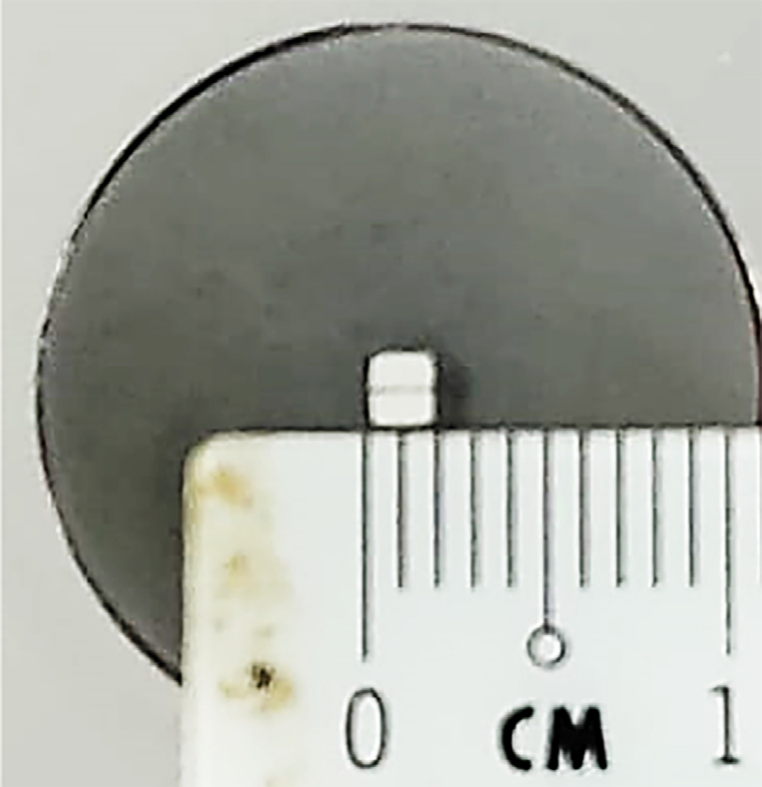
ATR size of INVENIO-R (2 mm).

As shown in Fig. 46, the FT-IR spectra were acquired using a Bruker Invenio-R (Universiti Putra Malaysia) spectrometer equipped with attenuated total reflection (ATR) (2 mm) diamond. The spectra acquisition involved 64 scans with a spectral resolution of 4 cm^−1^ and the spectra were processed using OPUS 8.7.41. With a temperature control unit, the temperature is maintained at room temperature ∼26  °C during the acquisition of the IR spectra. Before the IR spectra of the sample were acquired, acetone was wiped on the ATR to remove contaminants from the previous sample which evaporated to dryness. The background spectrum is collected, which will subtract any unwanted residual peaks from the sample spectrum and avoid the contaminants reading. Then, the samples spectra were recorded immediately on the ATR and analyzed by OPUS 8.7.41. The configurations and numerical values of the advanced parameters (such as resolution, sample scan time, background scan time, and spectral range) are saved [3].

Fig. 47 shows the configurations and numerical values of the advanced parameters (such as resolution, sample scan time, background scan time, and spectral range). Additionally, the phase resolution is stored within the Fourier transform and the optical parameters are also displayed under these experimental conditions in and Figs. 48 and 49, respectively (Fig. 1, Fig. 2, Fig. 3, Fig. 4, Fig. 5, Fig. 6, Fig. 7, Fig. 8, Fig. 9, Fig. 10, Fig. 11, Fig. 12, Fig. 13, Fig. 14, Fig. 15, Fig. 16, Fig. 17, Fig. 18, Fig. 19, Fig. 20, Fig. 21, Fig. 22, Fig. 23, Fig. 24, Fig. 25, Fig. 26, Fig. 27, Fig. 28, Fig. 29, Fig. 30, Fig. 31, Fig. 32, Fig. 33, Fig. 34, Fig. 35, Fig. 36, Fig. 37, Fig. 38, Fig. 39, Fig. 40, Fig. 41, Fig. 42, Fig. 43, Fig. 44, Fig. 45).

**Fig. 47:**
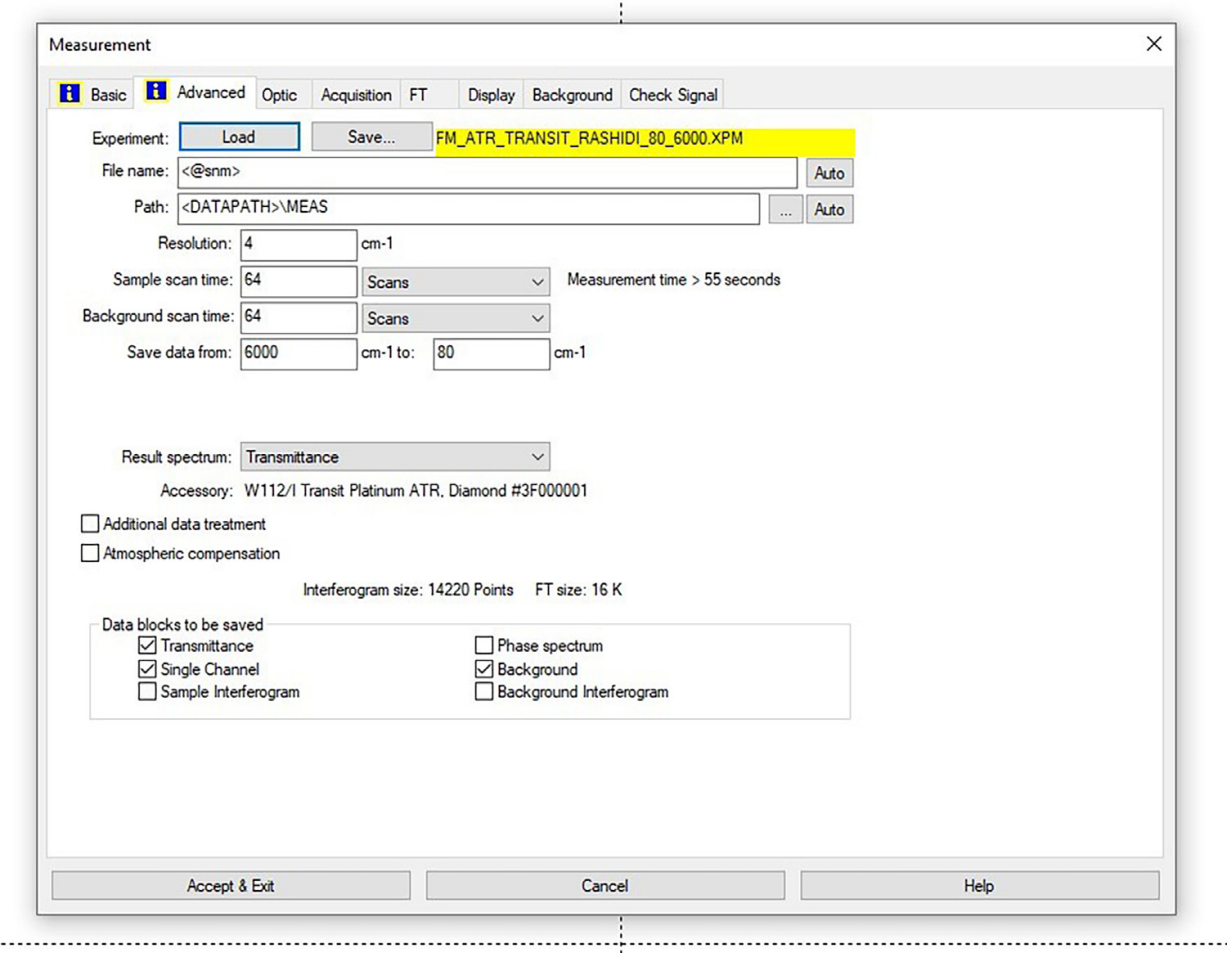
Configurations and numerical values of the advanced parameters (such as resolution, sample scan time, background scan time, and spectral range).

**Fig. 48:**
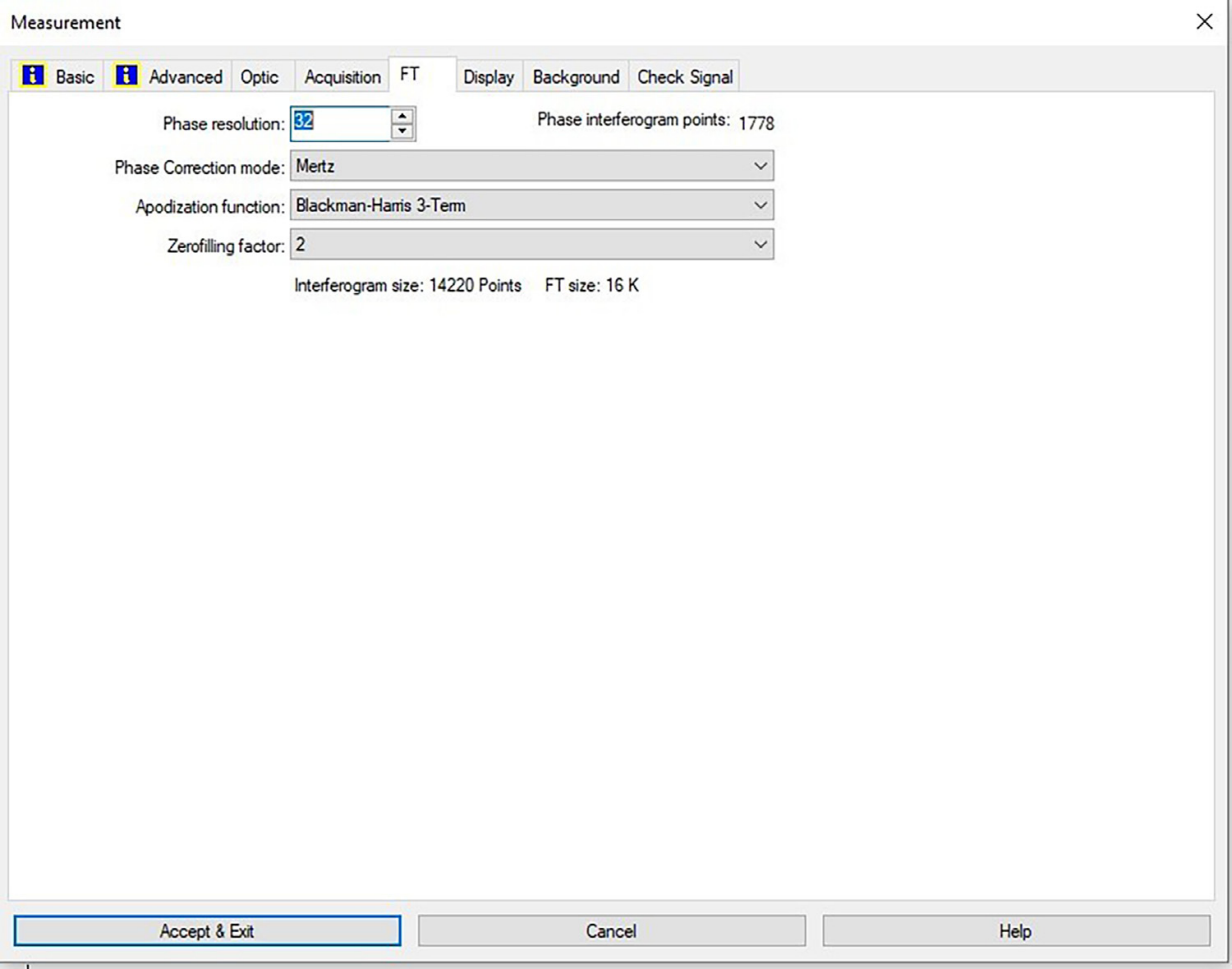
Configurations and numerical values of the advanced parameters of the phase resolution.

**Fig. 49:**
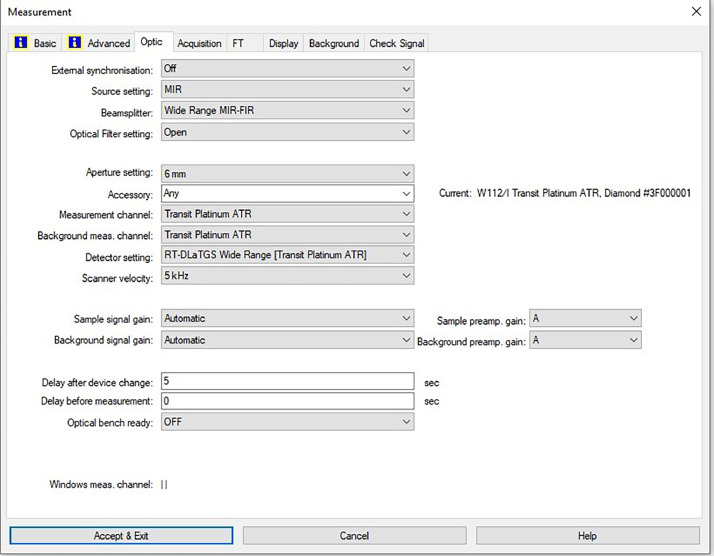
Configurations and numerical values of the optical parameters.

## Limitations

Not applicable.

## Ethics Statement

The work does not involve human subjects, animal experiments, or data collected from social media platforms.

## CRediT authorship contribution statement

**Mohd Rashidi Abdull Manap:** Conceptualization, Funding acquisition, Methodology, Writing – original draft, Supervision. **Haziq Izwan Baharim:** Methodology, Validation, Writing – original draft. **Nur Azalina Ajmahera Shamsudin:** Methodology, Software, Investigation, Visualization, Project administration. **Ahmad Faridi Ferdaus:** Writing – review & editing, Resources, Investigation.

## Data Availability

Experimental raw data of B7 and B10 (Original data) (Mendeley Data). Experimental data of crime-related fuel in Johor and Terengganu, Malaysia. (Original data) (Mendeley Data). Experiment files and measurement parameters for Bruker Invenio-R (Original data) (Mendeley Data).
